# 4-*tert*-Butyl-*N*-[(2,6-dimethyl­phen­yl)carbamothio­yl]benzamide

**DOI:** 10.1107/S1600536812034174

**Published:** 2012-08-11

**Authors:** M. Sukeri M. Yusof, Suhana Arshad, Ibrahim Abdul Razak, Azhar Abdul Rahman

**Affiliations:** a Department of Chemical Sciences, Faculty of Science and Technology, Universiti Malaysia Terengganu, Menggabang Telipot, 21030 Kuala Terengganu, Malaysia; b School of Physics, Universiti Sains Malaysia, 11800 USM, Penang, Malaysia

## Abstract

The asymmetric unit of the title compound, C_20_H_24_N_2_OS, consists of two crystallographically independent mol­ecules. In each mol­ecule, an intra­molecular N—H⋯O hydrogen bond forms an *S*(6) ring motif. The dihedral angles between the terminal benzene rings in the two mol­ecules are 75.52 (7) and 42.80 (7)°. In the crystal, inter­molecular N—H⋯S inter­actions link the mol­ecules into a chain along the *c* axis.

## Related literature

For related structures, see: Yusof, Mutalib *et al.* (2012 ▶); Yusof, Embong *et al.* (2012*a* ▶,*b* ▶); Usman *et al.* (2002 ▶); Al-abbasi *et al.* (2010 ▶). For hydrogen-bond motifs, see: Bernstein *et al.* (1995 ▶). For the stability of the temperature controller used in the data collection, see: Cosier & Glazer (1986 ▶).
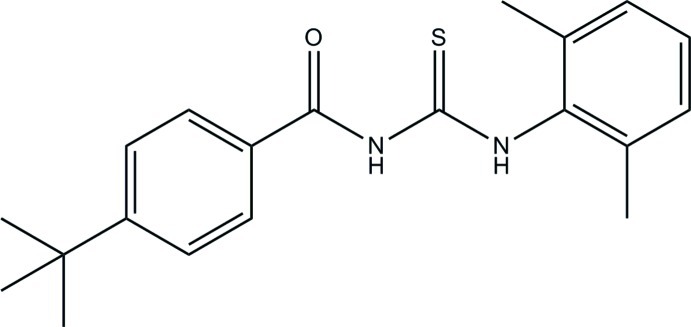


## Experimental

### 

#### Crystal data

C_20_H_24_N_2_OS
*M* *_r_* = 340.47
Monoclinic, 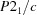

*a* = 19.5893 (2) Å
*b* = 8.8118 (1) Å
*c* = 23.5034 (2) Å
β = 114.886 (1)°
*V* = 3680.37 (6) Å^3^
*Z* = 8
Mo *K*α radiation
μ = 0.18 mm^−1^
*T* = 100 K
0.41 × 0.22 × 0.17 mm

#### Data collection

Bruker SMART APEXII CCD area-detector diffractometer
Absorption correction: multi-scan (*SADABS*; Bruker, 2009 ▶) *T*_min_ = 0.929, *T*_max_ = 0.970
67130 measured reflections
10823 independent reflections
8234 reflections with *I* > 2σ(*I*)
*R*_int_ = 0.044

#### Refinement

*R*[*F* ^2^ > 2σ(*F* ^2^)] = 0.051
*wR*(*F* ^2^) = 0.120
*S* = 1.04
10823 reflections
459 parameters
H atoms treated by a mixture of independent and constrained refinement
Δρ_max_ = 0.43 e Å^−3^
Δρ_min_ = −0.24 e Å^−3^

### 

Data collection: *APEX2* (Bruker, 2009 ▶); cell refinement: *SAINT* (Bruker, 2009 ▶); data reduction: *SAINT*; program(s) used to solve structure: *SHELXTL* (Sheldrick, 2008 ▶); program(s) used to refine structure: *SHELXTL*; molecular graphics: *SHELXTL*; software used to prepare material for publication: *SHELXTL* and *PLATON* (Spek, 2009 ▶).

## Supplementary Material

Crystal structure: contains datablock(s) global, I. DOI: 10.1107/S1600536812034174/is5176sup1.cif

Structure factors: contains datablock(s) I. DOI: 10.1107/S1600536812034174/is5176Isup2.hkl

Supplementary material file. DOI: 10.1107/S1600536812034174/is5176Isup3.cml

Additional supplementary materials:  crystallographic information; 3D view; checkCIF report

## Figures and Tables

**Table 1:** Hydrogen-bond geometry (Å, °)

*D*—H⋯*A*	*D*—H	H⋯*A*	*D*⋯*A*	*D*—H⋯*A*
N2*A*—H2*NA*⋯O1*A*	0.84 (2)	2.06 (2)	2.6972 (19)	133.1 (16)
N2*B*—H2*NB*⋯O1*B*	0.84 (2)	2.08 (2)	2.7183 (19)	132.4 (17)
N2*A*—H2*NA*⋯S1*B*	0.84 (2)	2.715 (17)	3.2598 (12)	124.3 (16)
N2*B*—H2*NB*⋯S1*A* ^i^	0.84 (2)	2.780 (19)	3.3044 (12)	121.9 (16)

Symmetry code: (i) 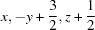.

**Fig. 1.**
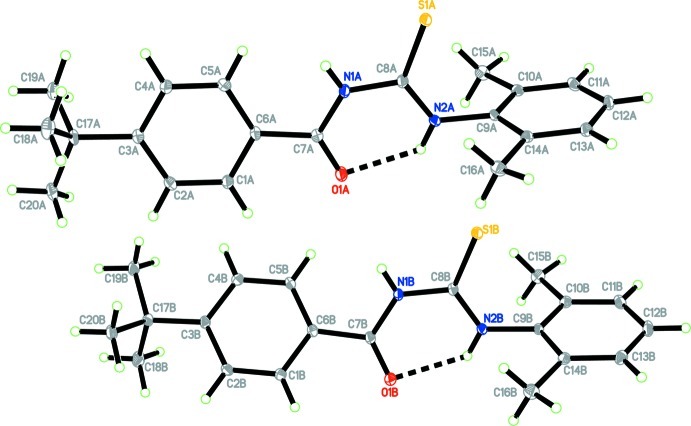
The molecular structure of the title compound with atom labels with 30% probability displacement ellipsoids.

**Fig. 2.**
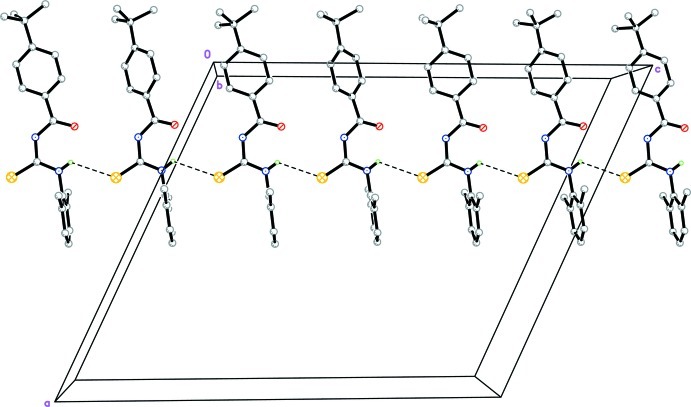
The crystal packing of the title compound. The H atoms not involved in the intermolecular interactions (dashed lines) have been omitted for clarity.

## supplementary crystallographic information

### Comment

In continuation of our work on synthesis of thiourea derivatives (Yusof, Mutalib *et al.*, 2012; Yusof, Embong *et al.*, 2012*a*,*b*) the title compound is prepared and its crystal structure is reported.

The asymmetric unit of the title compound consists of two crystallographically independent molecules *A* and *B* (Fig. 1). In both molecules, the intramolecular N2A—H2NA···O1A and N2B—H2NB···O1B hydrogen bonds (Table 1) generate *S*(6) ring motifs (Bernstein *et al.*, 1995). The dihedral angles between the two terminal benzene rings in molecule *A* and *B* are 75.52 (7) and 42.80 (7)°, respectively. The bond lengths and angles are within normal ranges and comparable to the previously reported structures (Usman *et al.*, 2002; Al-abbasi *et al.*, 2010).

The crystal packing is shown in Fig. 2. The intermolecular N2A—H2NA···S1B and N2B—H2NB···S1A interactions (Table 1) link the molecules into a one-dimensional chain along the *c* axis.

### Experimental

30 ml acetone solution of 2,4-dimethylaniline (0.93 g, 7.7 mmol) was added into 30 ml acetone containing 4-*tert*-butylbenzoyl chloride (1.50 g, 7.7 mmol) and ammonium thiocyanate (0.58 g, 9.5 mmol). The mixture was refluxed for 2.5 hours. The solution was filtered and left to evaporate at room temperature. The yellowish precipitate obtained after a few days was washed with water and cold ethanol. The crystals were obtained by recrystallization from DMF.

### Refinement

N-bound H atoms were located from a difference map and refined freely [N—H = 0.813 (19)–0.84 (2) Å]. The remaining H atoms were positioned geometrically (C—H = 0.93 or 0.96 Å) and refined using a riding model with *U*_iso_(H) = 1.2 or 1.5*U*_eq_(C). A rotating group model was applied to the methyl groups.

### Crystal data

**Table:**

C_20_H_24_N_2_OS	*F*(000) = 1456
*M**_r_* = 340.47	*D*_x_ = 1.229 Mg m^−^^3^
Monoclinic, *P*2_1_/*c*	Mo *K*α radiation, λ = 0.71073 Å
Hall symbol: -P 2ybc	Cell parameters from 9861 reflections
*a* = 19.5893 (2) Å	θ = 2.3–30.0°
*b* = 8.8118 (1) Å	µ = 0.18 mm^−^^1^
*c* = 23.5034 (2) Å	*T* = 100 K
β = 114.886 (1)°	Block, yellow
*V* = 3680.37 (6) Å^3^	0.41 × 0.22 × 0.17 mm
*Z* = 8	

### Data collection

**Table:**

Bruker SMART APEXII CCD area-detector diffractometer	10823 independent reflections
Radiation source: fine-focus sealed tube	8234 reflections with *I* > 2σ(*I*)
Graphite monochromator	*R*_int_ = 0.044
φ and ω scans	θ_max_ = 30.1°, θ_min_ = 1.8°
Absorption correction: multi-scan (*SADABS*; Bruker, 2009)	*h* = −27→27
*T*_min_ = 0.929, *T*_max_ = 0.970	*k* = −12→12
67130 measured reflections	*l* = −33→33

### Refinement

**Table:**

Refinement on *F*^2^	Primary atom site location: structure-invariant direct methods
Least-squares matrix: full	Secondary atom site location: difference Fourier map
*R*[*F*^2^ > 2σ(*F*^2^)] = 0.051	Hydrogen site location: inferred from neighbouring sites
*wR*(*F*^2^) = 0.120	H atoms treated by a mixture of independent and constrained refinement
*S* = 1.04	*w* = 1/[σ^2^(*F*_o_^2^) + (0.0433*P*)^2^ + 2.0471*P*] where *P* = (*F*_o_^2^ + 2*F*_c_^2^)/3
10823 reflections	(Δ/σ)_max_ = 0.001
459 parameters	Δρ_max_ = 0.43 e Å^−^^3^
0 restraints	Δρ_min_ = −0.24 e Å^−^^3^

### Special details

**Table:**

Experimental. The crystal was placed in the cold stream of an Oxford Cryosystems Cobra open-flow nitrogen cryostat (Cosier & Glazer, 1986) operating at 100.0 (1) K.
Geometry. All e.s.d.'s (except the e.s.d. in the dihedral angle between two l.s. planes) are estimated using the full covariance matrix. The cell e.s.d.'s are taken into account individually in the estimation of e.s.d.'s in distances, angles and torsion angles; correlations between e.s.d.'s in cell parameters are only used when they are defined by crystal symmetry. An approximate (isotropic) treatment of cell e.s.d.'s is used for estimating e.s.d.'s involving l.s. planes.
Refinement. Refinement of *F*^2^ against ALL reflections. The weighted *R*-factor *wR* and goodness of fit *S* are based on *F*^2^, conventional *R*-factors *R* are based on *F*, with *F* set to zero for negative *F*^2^. The threshold expression of *F*^2^ > σ(*F*^2^) is used only for calculating *R*-factors(gt) *etc*. and is not relevant to the choice of reflections for refinement. *R*-factors based on *F*^2^ are statistically about twice as large as those based on *F*, and *R*- factors based on ALL data will be even larger.

### Fractional atomic coordinates and isotropic or equivalent isotropic displacement parameters (Å^2^)

**Table:**

	*x*	*y*	*z*	*U*_iso_*/*U*_eq_	
S1A	0.33613 (2)	0.71302 (5)	0.125254 (16)	0.02173 (9)	
O1A	0.17289 (6)	0.71337 (13)	0.21821 (5)	0.0218 (2)	
N1A	0.20809 (7)	0.71907 (15)	0.13716 (6)	0.0175 (2)	
N2A	0.31610 (7)	0.70427 (14)	0.23058 (5)	0.0151 (2)	
C1A	0.02289 (8)	0.77443 (18)	0.13710 (7)	0.0205 (3)	
H1AA	0.0382	0.7907	0.1798	0.025*	
C2A	−0.05243 (9)	0.79149 (19)	0.09627 (7)	0.0222 (3)	
H2AA	−0.0867	0.8200	0.1122	0.027*	
C3A	−0.07793 (8)	0.76693 (16)	0.03200 (7)	0.0170 (3)	
C4A	−0.02428 (9)	0.72404 (19)	0.01032 (7)	0.0229 (3)	
H4AA	−0.0397	0.7062	−0.0323	0.028*	
C5A	0.05104 (9)	0.70740 (18)	0.05048 (7)	0.0222 (3)	
H5AA	0.0854	0.6789	0.0346	0.027*	
C6A	0.07568 (8)	0.73333 (16)	0.11479 (6)	0.0159 (3)	
C7A	0.15550 (8)	0.72049 (16)	0.16165 (6)	0.0160 (3)	
C8A	0.28677 (8)	0.71169 (16)	0.16839 (6)	0.0157 (3)	
C9A	0.39627 (8)	0.69750 (16)	0.26710 (6)	0.0136 (3)	
C10A	0.43051 (8)	0.55544 (16)	0.28225 (6)	0.0154 (3)	
C11A	0.50857 (8)	0.55097 (17)	0.31649 (7)	0.0188 (3)	
H11A	0.5332	0.4580	0.3270	0.023*	
C12A	0.54946 (8)	0.68438 (18)	0.33479 (7)	0.0199 (3)	
H12A	0.6015	0.6799	0.3570	0.024*	
C13A	0.51391 (8)	0.82473 (17)	0.32053 (7)	0.0182 (3)	
H13A	0.5421	0.9132	0.3338	0.022*	
C14A	0.43598 (8)	0.83310 (16)	0.28633 (6)	0.0150 (3)	
C15A	0.38419 (9)	0.41307 (17)	0.26297 (7)	0.0222 (3)	
H15A	0.3561	0.4120	0.2182	0.033*	
H15B	0.3500	0.4100	0.2826	0.033*	
H15C	0.4168	0.3263	0.2757	0.033*	
C16A	0.39558 (9)	0.98275 (17)	0.27133 (7)	0.0213 (3)	
H16A	0.3661	0.9904	0.2269	0.032*	
H16B	0.4317	1.0638	0.2850	0.032*	
H16C	0.3632	0.9898	0.2925	0.032*	
C17A	−0.16009 (8)	0.79010 (17)	−0.01444 (7)	0.0199 (3)	
C18A	−0.16415 (10)	0.9314 (2)	−0.05418 (8)	0.0301 (4)	
H18A	−0.1473	1.0186	−0.0273	0.045*	
H18B	−0.2151	0.9468	−0.0842	0.045*	
H18C	−0.1325	0.9169	−0.0757	0.045*	
C19A	−0.18877 (9)	0.65003 (19)	−0.05713 (7)	0.0248 (3)	
H19A	−0.1865	0.5626	−0.0320	0.037*	
H19B	−0.1578	0.6335	−0.0792	0.037*	
H19C	−0.2399	0.6667	−0.0868	0.037*	
C20A	−0.21171 (9)	0.8148 (2)	0.01879 (8)	0.0313 (4)	
H20A	−0.1971	0.9059	0.0434	0.047*	
H20B	−0.2075	0.7299	0.0457	0.047*	
H20C	−0.2628	0.8240	−0.0119	0.047*	
S1B	0.33713 (2)	0.71970 (5)	0.375613 (16)	0.01977 (9)	
O1B	0.16877 (6)	0.80776 (13)	0.45954 (5)	0.0227 (2)	
N1B	0.20713 (7)	0.73436 (14)	0.38416 (6)	0.0156 (2)	
N2B	0.31391 (7)	0.76209 (14)	0.47819 (5)	0.0151 (2)	
C1B	0.01921 (8)	0.84843 (17)	0.36482 (7)	0.0188 (3)	
H1BA	0.0335	0.9178	0.3976	0.023*	
C2B	−0.05593 (8)	0.83488 (17)	0.32340 (7)	0.0194 (3)	
H2BA	−0.0911	0.8985	0.3280	0.023*	
C3B	−0.08026 (8)	0.72766 (16)	0.27467 (7)	0.0159 (3)	
C4B	−0.02515 (8)	0.64045 (17)	0.26716 (7)	0.0196 (3)	
H4BA	−0.0393	0.5703	0.2346	0.024*	
C5B	0.05073 (8)	0.65653 (17)	0.30748 (7)	0.0188 (3)	
H5BA	0.0865	0.5987	0.3009	0.023*	
C6B	0.07355 (8)	0.75798 (16)	0.35734 (7)	0.0156 (3)	
C7B	0.15330 (8)	0.76964 (16)	0.40537 (7)	0.0159 (3)	
C8B	0.28580 (8)	0.74046 (15)	0.41641 (6)	0.0147 (3)	
C9B	0.39374 (8)	0.77562 (16)	0.51574 (6)	0.0144 (3)	
C10B	0.43690 (8)	0.64373 (16)	0.53508 (6)	0.0169 (3)	
C11B	0.51411 (9)	0.66015 (18)	0.57088 (7)	0.0212 (3)	
H11B	0.5444	0.5745	0.5845	0.025*	
C12B	0.54591 (9)	0.80337 (19)	0.58635 (7)	0.0231 (3)	
H12B	0.5976	0.8129	0.6097	0.028*	
C13B	0.50172 (9)	0.93235 (18)	0.56754 (7)	0.0212 (3)	
H13B	0.5239	1.0276	0.5786	0.025*	
C14B	0.42422 (8)	0.92070 (16)	0.53205 (6)	0.0168 (3)	
C15B	0.40121 (9)	0.48993 (17)	0.51972 (7)	0.0230 (3)	
H15D	0.4396	0.4134	0.5345	0.035*	
H15E	0.3734	0.4807	0.4751	0.035*	
H15F	0.3678	0.4772	0.5397	0.035*	
C16B	0.37459 (9)	1.05825 (18)	0.51222 (7)	0.0240 (3)	
H16D	0.3453	1.0560	0.4676	0.036*	
H16E	0.4051	1.1481	0.5234	0.036*	
H16F	0.3415	1.0587	0.5329	0.036*	
C17B	−0.16468 (8)	0.70816 (17)	0.23318 (7)	0.0178 (3)	
C18B	−0.20564 (9)	0.6758 (2)	0.27498 (8)	0.0258 (3)	
H18D	−0.1854	0.5855	0.2991	0.039*	
H18E	−0.2583	0.6617	0.2492	0.039*	
H18F	−0.1989	0.7599	0.3028	0.039*	
C19B	−0.18011 (9)	0.57519 (18)	0.18737 (7)	0.0244 (3)	
H19D	−0.1609	0.4832	0.2105	0.037*	
H19E	−0.1557	0.5935	0.1601	0.037*	
H19F	−0.2334	0.5655	0.1628	0.037*	
C20B	−0.19667 (9)	0.85460 (18)	0.19550 (7)	0.0218 (3)	
H20D	−0.2496	0.8423	0.1702	0.033*	
H20E	−0.1716	0.8748	0.1689	0.033*	
H20F	−0.1888	0.9379	0.2239	0.033*	
H2NA	0.2879 (10)	0.707 (2)	0.2491 (8)	0.022 (5)*	
H2NB	0.2845 (11)	0.776 (2)	0.4955 (9)	0.031 (5)*	
H1NB	0.1927 (11)	0.720 (2)	0.3461 (9)	0.027 (5)*	
H1NA	0.1930 (11)	0.727 (2)	0.0994 (9)	0.030 (5)*	

### Atomic displacement parameters (Å^2^)

**Table:**

	*U*^11^	*U*^22^	*U*^33^	*U*^12^	*U*^13^	*U*^23^
S1A	0.01282 (18)	0.0403 (2)	0.01273 (16)	−0.00115 (15)	0.00602 (14)	−0.00031 (14)
O1A	0.0139 (5)	0.0350 (6)	0.0162 (5)	0.0015 (4)	0.0062 (4)	0.0028 (4)
N1A	0.0114 (6)	0.0274 (6)	0.0124 (5)	0.0003 (5)	0.0036 (5)	0.0013 (5)
N2A	0.0121 (6)	0.0216 (6)	0.0130 (5)	0.0010 (5)	0.0069 (5)	0.0005 (4)
C1A	0.0135 (7)	0.0320 (8)	0.0155 (6)	0.0011 (6)	0.0056 (6)	0.0001 (6)
C2A	0.0149 (7)	0.0319 (8)	0.0213 (7)	0.0031 (6)	0.0092 (6)	−0.0011 (6)
C3A	0.0132 (7)	0.0184 (7)	0.0184 (7)	0.0003 (5)	0.0055 (6)	0.0018 (5)
C4A	0.0150 (8)	0.0355 (9)	0.0172 (7)	0.0002 (6)	0.0056 (6)	−0.0033 (6)
C5A	0.0157 (7)	0.0317 (8)	0.0203 (7)	0.0011 (6)	0.0088 (6)	−0.0039 (6)
C6A	0.0117 (7)	0.0180 (6)	0.0166 (6)	0.0001 (5)	0.0046 (5)	0.0016 (5)
C7A	0.0141 (7)	0.0162 (6)	0.0173 (6)	0.0007 (5)	0.0063 (5)	0.0013 (5)
C8A	0.0125 (7)	0.0182 (6)	0.0156 (6)	−0.0005 (5)	0.0051 (5)	0.0002 (5)
C9A	0.0107 (6)	0.0195 (7)	0.0109 (6)	0.0013 (5)	0.0048 (5)	0.0009 (5)
C10A	0.0161 (7)	0.0170 (6)	0.0150 (6)	0.0009 (5)	0.0086 (5)	0.0011 (5)
C11A	0.0166 (7)	0.0222 (7)	0.0185 (7)	0.0069 (5)	0.0083 (6)	0.0053 (5)
C12A	0.0113 (7)	0.0308 (8)	0.0158 (6)	0.0023 (6)	0.0041 (5)	0.0022 (5)
C13A	0.0154 (7)	0.0230 (7)	0.0166 (6)	−0.0034 (5)	0.0071 (6)	−0.0015 (5)
C14A	0.0149 (7)	0.0173 (6)	0.0137 (6)	0.0012 (5)	0.0069 (5)	0.0001 (5)
C15A	0.0235 (8)	0.0179 (7)	0.0267 (8)	−0.0016 (6)	0.0119 (7)	0.0002 (6)
C16A	0.0227 (8)	0.0182 (7)	0.0236 (7)	0.0037 (6)	0.0105 (6)	0.0017 (5)
C17A	0.0116 (7)	0.0243 (7)	0.0204 (7)	0.0030 (5)	0.0034 (6)	0.0032 (6)
C18A	0.0241 (9)	0.0289 (9)	0.0285 (8)	0.0018 (7)	0.0025 (7)	0.0081 (7)
C19A	0.0150 (7)	0.0289 (8)	0.0250 (8)	−0.0023 (6)	0.0030 (6)	−0.0007 (6)
C20A	0.0121 (8)	0.0489 (11)	0.0294 (8)	0.0061 (7)	0.0055 (7)	−0.0008 (7)
S1B	0.01337 (18)	0.0342 (2)	0.01263 (15)	0.00004 (14)	0.00637 (14)	−0.00061 (14)
O1B	0.0147 (5)	0.0346 (6)	0.0181 (5)	−0.0001 (4)	0.0063 (4)	−0.0059 (4)
N1B	0.0102 (6)	0.0230 (6)	0.0125 (5)	0.0005 (4)	0.0037 (5)	−0.0006 (4)
N2B	0.0110 (6)	0.0221 (6)	0.0131 (5)	−0.0007 (4)	0.0059 (5)	−0.0011 (4)
C1B	0.0145 (7)	0.0207 (7)	0.0201 (7)	−0.0021 (5)	0.0064 (6)	−0.0059 (5)
C2B	0.0138 (7)	0.0204 (7)	0.0242 (7)	0.0005 (5)	0.0082 (6)	−0.0055 (6)
C3B	0.0116 (7)	0.0167 (6)	0.0181 (6)	−0.0018 (5)	0.0050 (5)	−0.0007 (5)
C4B	0.0162 (7)	0.0214 (7)	0.0197 (7)	−0.0008 (5)	0.0061 (6)	−0.0058 (5)
C5B	0.0132 (7)	0.0215 (7)	0.0206 (7)	0.0029 (5)	0.0060 (6)	−0.0031 (5)
C6B	0.0116 (7)	0.0173 (6)	0.0181 (6)	−0.0005 (5)	0.0063 (5)	0.0006 (5)
C7B	0.0116 (7)	0.0173 (6)	0.0185 (6)	−0.0002 (5)	0.0059 (5)	−0.0001 (5)
C8B	0.0115 (7)	0.0164 (6)	0.0157 (6)	0.0000 (5)	0.0054 (5)	0.0013 (5)
C9B	0.0114 (7)	0.0219 (7)	0.0110 (6)	−0.0014 (5)	0.0057 (5)	−0.0005 (5)
C10B	0.0169 (7)	0.0209 (7)	0.0137 (6)	−0.0009 (5)	0.0073 (5)	0.0003 (5)
C11B	0.0163 (7)	0.0300 (8)	0.0177 (7)	0.0044 (6)	0.0076 (6)	0.0040 (6)
C12B	0.0122 (7)	0.0390 (9)	0.0167 (7)	−0.0034 (6)	0.0048 (6)	0.0011 (6)
C13B	0.0190 (8)	0.0286 (8)	0.0171 (7)	−0.0086 (6)	0.0086 (6)	−0.0036 (6)
C14B	0.0159 (7)	0.0218 (7)	0.0145 (6)	−0.0020 (5)	0.0083 (5)	−0.0013 (5)
C15B	0.0248 (8)	0.0207 (7)	0.0217 (7)	−0.0001 (6)	0.0079 (6)	0.0005 (6)
C16B	0.0253 (8)	0.0218 (7)	0.0256 (8)	0.0003 (6)	0.0114 (7)	0.0004 (6)
C17B	0.0123 (7)	0.0196 (7)	0.0190 (6)	−0.0025 (5)	0.0041 (5)	−0.0026 (5)
C18B	0.0164 (8)	0.0352 (9)	0.0252 (8)	−0.0066 (6)	0.0081 (6)	−0.0002 (6)
C19B	0.0177 (8)	0.0244 (8)	0.0249 (8)	−0.0022 (6)	0.0028 (6)	−0.0061 (6)
C20B	0.0152 (7)	0.0241 (7)	0.0230 (7)	0.0003 (6)	0.0051 (6)	−0.0005 (6)

### Geometric parameters (Å, º)

**Table:**

S1A—C8A	1.6697 (15)	S1B—C8B	1.6659 (15)
O1A—C7A	1.2273 (17)	O1B—C7B	1.2249 (17)
N1A—C7A	1.3754 (19)	N1B—C7B	1.3784 (19)
N1A—C8A	1.4021 (18)	N1B—C8B	1.4033 (18)
N1A—H1NA	0.813 (19)	N1B—H1NB	0.826 (19)
N2A—C8A	1.3282 (17)	N2B—C8B	1.3323 (17)
N2A—C9A	1.4396 (18)	N2B—C9B	1.4412 (18)
N2A—H2NA	0.836 (19)	N2B—H2NB	0.84 (2)
C1A—C2A	1.389 (2)	C1B—C2B	1.387 (2)
C1A—C6A	1.390 (2)	C1B—C6B	1.399 (2)
C1A—H1AA	0.9300	C1B—H1BA	0.9300
C2A—C3A	1.394 (2)	C2B—C3B	1.4043 (19)
C2A—H2AA	0.9300	C2B—H2BA	0.9300
C3A—C4A	1.399 (2)	C3B—C4B	1.395 (2)
C3A—C17A	1.531 (2)	C3B—C17B	1.5356 (19)
C4A—C5A	1.385 (2)	C4B—C5B	1.394 (2)
C4A—H4AA	0.9300	C4B—H4BA	0.9300
C5A—C6A	1.399 (2)	C5B—C6B	1.3898 (19)
C5A—H5AA	0.9300	C5B—H5BA	0.9300
C6A—C7A	1.4905 (19)	C6B—C7B	1.4970 (19)
C9A—C14A	1.3933 (19)	C9B—C14B	1.395 (2)
C9A—C10A	1.3938 (19)	C9B—C10B	1.396 (2)
C10A—C11A	1.397 (2)	C10B—C11B	1.395 (2)
C10A—C15A	1.502 (2)	C10B—C15B	1.498 (2)
C11A—C12A	1.385 (2)	C11B—C12B	1.387 (2)
C11A—H11A	0.9300	C11B—H11B	0.9300
C12A—C13A	1.389 (2)	C12B—C13B	1.384 (2)
C12A—H12A	0.9300	C12B—H12B	0.9300
C13A—C14A	1.396 (2)	C13B—C14B	1.395 (2)
C13A—H13A	0.9300	C13B—H13B	0.9300
C14A—C16A	1.501 (2)	C14B—C16B	1.500 (2)
C15A—H15A	0.9600	C15B—H15D	0.9600
C15A—H15B	0.9600	C15B—H15E	0.9600
C15A—H15C	0.9600	C15B—H15F	0.9600
C16A—H16A	0.9600	C16B—H16D	0.9600
C16A—H16B	0.9600	C16B—H16E	0.9600
C16A—H16C	0.9600	C16B—H16F	0.9600
C17A—C20A	1.531 (2)	C17B—C19B	1.533 (2)
C17A—C18A	1.538 (2)	C17B—C18B	1.534 (2)
C17A—C19A	1.540 (2)	C17B—C20B	1.541 (2)
C18A—H18A	0.9600	C18B—H18D	0.9600
C18A—H18B	0.9600	C18B—H18E	0.9600
C18A—H18C	0.9600	C18B—H18F	0.9600
C19A—H19A	0.9600	C19B—H19D	0.9600
C19A—H19B	0.9600	C19B—H19E	0.9600
C19A—H19C	0.9600	C19B—H19F	0.9600
C20A—H20A	0.9600	C20B—H20D	0.9600
C20A—H20B	0.9600	C20B—H20E	0.9600
C20A—H20C	0.9600	C20B—H20F	0.9600
			
C7A—N1A—C8A	129.29 (12)	C7B—N1B—C8B	129.01 (12)
C7A—N1A—H1NA	117.7 (14)	C7B—N1B—H1NB	117.4 (13)
C8A—N1A—H1NA	112.9 (14)	C8B—N1B—H1NB	112.9 (13)
C8A—N2A—C9A	121.09 (12)	C8B—N2B—C9B	121.58 (12)
C8A—N2A—H2NA	119.9 (12)	C8B—N2B—H2NB	119.7 (13)
C9A—N2A—H2NA	119.0 (12)	C9B—N2B—H2NB	118.5 (13)
C2A—C1A—C6A	120.68 (14)	C2B—C1B—C6B	120.13 (13)
C2A—C1A—H1AA	119.7	C2B—C1B—H1BA	119.9
C6A—C1A—H1AA	119.7	C6B—C1B—H1BA	119.9
C1A—C2A—C3A	121.60 (14)	C1B—C2B—C3B	121.75 (14)
C1A—C2A—H2AA	119.2	C1B—C2B—H2BA	119.1
C3A—C2A—H2AA	119.2	C3B—C2B—H2BA	119.1
C2A—C3A—C4A	117.07 (13)	C4B—C3B—C2B	117.29 (13)
C2A—C3A—C17A	122.80 (13)	C4B—C3B—C17B	122.80 (13)
C4A—C3A—C17A	120.10 (13)	C2B—C3B—C17B	119.90 (13)
C5A—C4A—C3A	121.91 (14)	C5B—C4B—C3B	121.24 (13)
C5A—C4A—H4AA	119.0	C5B—C4B—H4BA	119.4
C3A—C4A—H4AA	119.0	C3B—C4B—H4BA	119.4
C4A—C5A—C6A	120.23 (14)	C6B—C5B—C4B	120.75 (13)
C4A—C5A—H5AA	119.9	C6B—C5B—H5BA	119.6
C6A—C5A—H5AA	119.9	C4B—C5B—H5BA	119.6
C1A—C6A—C5A	118.50 (13)	C5B—C6B—C1B	118.73 (13)
C1A—C6A—C7A	117.18 (13)	C5B—C6B—C7B	122.92 (13)
C5A—C6A—C7A	124.32 (13)	C1B—C6B—C7B	118.29 (13)
O1A—C7A—N1A	122.52 (13)	O1B—C7B—N1B	123.05 (13)
O1A—C7A—C6A	122.06 (13)	O1B—C7B—C6B	121.73 (13)
N1A—C7A—C6A	115.41 (12)	N1B—C7B—C6B	115.23 (12)
N2A—C8A—N1A	116.70 (12)	N2B—C8B—N1B	116.77 (12)
N2A—C8A—S1A	125.15 (11)	N2B—C8B—S1B	124.77 (11)
N1A—C8A—S1A	118.15 (10)	N1B—C8B—S1B	118.46 (10)
C14A—C9A—C10A	122.99 (13)	C14B—C9B—C10B	122.90 (13)
C14A—C9A—N2A	118.55 (12)	C14B—C9B—N2B	118.22 (13)
C10A—C9A—N2A	118.46 (12)	C10B—C9B—N2B	118.88 (12)
C9A—C10A—C11A	117.70 (13)	C11B—C10B—C9B	117.67 (14)
C9A—C10A—C15A	120.56 (13)	C11B—C10B—C15B	121.13 (14)
C11A—C10A—C15A	121.73 (13)	C9B—C10B—C15B	121.17 (13)
C12A—C11A—C10A	120.31 (13)	C12B—C11B—C10B	120.40 (14)
C12A—C11A—H11A	119.8	C12B—C11B—H11B	119.8
C10A—C11A—H11A	119.8	C10B—C11B—H11B	119.8
C11A—C12A—C13A	120.99 (14)	C13B—C12B—C11B	120.81 (14)
C11A—C12A—H12A	119.5	C13B—C12B—H12B	119.6
C13A—C12A—H12A	119.5	C11B—C12B—H12B	119.6
C12A—C13A—C14A	120.10 (14)	C12B—C13B—C14B	120.52 (14)
C12A—C13A—H13A	119.9	C12B—C13B—H13B	119.7
C14A—C13A—H13A	119.9	C14B—C13B—H13B	119.7
C9A—C14A—C13A	117.86 (13)	C9B—C14B—C13B	117.66 (14)
C9A—C14A—C16A	120.66 (13)	C9B—C14B—C16B	120.58 (13)
C13A—C14A—C16A	121.47 (13)	C13B—C14B—C16B	121.76 (14)
C10A—C15A—H15A	109.5	C10B—C15B—H15D	109.5
C10A—C15A—H15B	109.5	C10B—C15B—H15E	109.5
H15A—C15A—H15B	109.5	H15D—C15B—H15E	109.5
C10A—C15A—H15C	109.5	C10B—C15B—H15F	109.5
H15A—C15A—H15C	109.5	H15D—C15B—H15F	109.5
H15B—C15A—H15C	109.5	H15E—C15B—H15F	109.5
C14A—C16A—H16A	109.5	C14B—C16B—H16D	109.5
C14A—C16A—H16B	109.5	C14B—C16B—H16E	109.5
H16A—C16A—H16B	109.5	H16D—C16B—H16E	109.5
C14A—C16A—H16C	109.5	C14B—C16B—H16F	109.5
H16A—C16A—H16C	109.5	H16D—C16B—H16F	109.5
H16B—C16A—H16C	109.5	H16E—C16B—H16F	109.5
C20A—C17A—C3A	112.14 (13)	C19B—C17B—C18B	107.82 (13)
C20A—C17A—C18A	108.56 (14)	C19B—C17B—C3B	111.90 (12)
C3A—C17A—C18A	108.12 (13)	C18B—C17B—C3B	109.13 (12)
C20A—C17A—C19A	107.93 (13)	C19B—C17B—C20B	108.92 (12)
C3A—C17A—C19A	110.09 (12)	C18B—C17B—C20B	109.14 (13)
C18A—C17A—C19A	109.98 (13)	C3B—C17B—C20B	109.88 (12)
C17A—C18A—H18A	109.5	C17B—C18B—H18D	109.5
C17A—C18A—H18B	109.5	C17B—C18B—H18E	109.5
H18A—C18A—H18B	109.5	H18D—C18B—H18E	109.5
C17A—C18A—H18C	109.5	C17B—C18B—H18F	109.5
H18A—C18A—H18C	109.5	H18D—C18B—H18F	109.5
H18B—C18A—H18C	109.5	H18E—C18B—H18F	109.5
C17A—C19A—H19A	109.5	C17B—C19B—H19D	109.5
C17A—C19A—H19B	109.5	C17B—C19B—H19E	109.5
H19A—C19A—H19B	109.5	H19D—C19B—H19E	109.5
C17A—C19A—H19C	109.5	C17B—C19B—H19F	109.5
H19A—C19A—H19C	109.5	H19D—C19B—H19F	109.5
H19B—C19A—H19C	109.5	H19E—C19B—H19F	109.5
C17A—C20A—H20A	109.5	C17B—C20B—H20D	109.5
C17A—C20A—H20B	109.5	C17B—C20B—H20E	109.5
H20A—C20A—H20B	109.5	H20D—C20B—H20E	109.5
C17A—C20A—H20C	109.5	C17B—C20B—H20F	109.5
H20A—C20A—H20C	109.5	H20D—C20B—H20F	109.5
H20B—C20A—H20C	109.5	H20E—C20B—H20F	109.5
			
C6A—C1A—C2A—C3A	0.6 (2)	C6B—C1B—C2B—C3B	2.4 (2)
C1A—C2A—C3A—C4A	0.1 (2)	C1B—C2B—C3B—C4B	−3.6 (2)
C1A—C2A—C3A—C17A	−177.84 (15)	C1B—C2B—C3B—C17B	175.19 (14)
C2A—C3A—C4A—C5A	−0.4 (2)	C2B—C3B—C4B—C5B	1.7 (2)
C17A—C3A—C4A—C5A	177.56 (15)	C17B—C3B—C4B—C5B	−177.09 (14)
C3A—C4A—C5A—C6A	0.1 (2)	C3B—C4B—C5B—C6B	1.4 (2)
C2A—C1A—C6A—C5A	−0.9 (2)	C4B—C5B—C6B—C1B	−2.7 (2)
C2A—C1A—C6A—C7A	178.83 (14)	C4B—C5B—C6B—C7B	174.29 (14)
C4A—C5A—C6A—C1A	0.6 (2)	C2B—C1B—C6B—C5B	0.8 (2)
C4A—C5A—C6A—C7A	−179.15 (14)	C2B—C1B—C6B—C7B	−176.34 (13)
C8A—N1A—C7A—O1A	−0.8 (2)	C8B—N1B—C7B—O1B	−3.1 (2)
C8A—N1A—C7A—C6A	178.44 (13)	C8B—N1B—C7B—C6B	177.00 (13)
C1A—C6A—C7A—O1A	15.5 (2)	C5B—C6B—C7B—O1B	−149.96 (15)
C5A—C6A—C7A—O1A	−164.77 (15)	C1B—C6B—C7B—O1B	27.0 (2)
C1A—C6A—C7A—N1A	−163.75 (13)	C5B—C6B—C7B—N1B	29.9 (2)
C5A—C6A—C7A—N1A	16.0 (2)	C1B—C6B—C7B—N1B	−153.11 (13)
C9A—N2A—C8A—N1A	−179.51 (12)	C9B—N2B—C8B—N1B	−177.78 (12)
C9A—N2A—C8A—S1A	0.4 (2)	C9B—N2B—C8B—S1B	2.5 (2)
C7A—N1A—C8A—N2A	0.3 (2)	C7B—N1B—C8B—N2B	10.6 (2)
C7A—N1A—C8A—S1A	−179.62 (12)	C7B—N1B—C8B—S1B	−169.63 (12)
C8A—N2A—C9A—C14A	89.39 (16)	C8B—N2B—C9B—C14B	98.87 (16)
C8A—N2A—C9A—C10A	−91.38 (16)	C8B—N2B—C9B—C10B	−82.20 (17)
C14A—C9A—C10A—C11A	−2.2 (2)	C14B—C9B—C10B—C11B	−1.7 (2)
N2A—C9A—C10A—C11A	178.57 (12)	N2B—C9B—C10B—C11B	179.41 (12)
C14A—C9A—C10A—C15A	176.84 (13)	C14B—C9B—C10B—C15B	176.33 (14)
N2A—C9A—C10A—C15A	−2.36 (19)	N2B—C9B—C10B—C15B	−2.5 (2)
C9A—C10A—C11A—C12A	0.6 (2)	C9B—C10B—C11B—C12B	0.1 (2)
C15A—C10A—C11A—C12A	−178.50 (14)	C15B—C10B—C11B—C12B	−177.96 (14)
C10A—C11A—C12A—C13A	1.1 (2)	C10B—C11B—C12B—C13B	1.1 (2)
C11A—C12A—C13A—C14A	−1.1 (2)	C11B—C12B—C13B—C14B	−0.6 (2)
C10A—C9A—C14A—C13A	2.2 (2)	C10B—C9B—C14B—C13B	2.1 (2)
N2A—C9A—C14A—C13A	−178.61 (12)	N2B—C9B—C14B—C13B	−178.98 (12)
C10A—C9A—C14A—C16A	−176.94 (13)	C10B—C9B—C14B—C16B	−177.36 (13)
N2A—C9A—C14A—C16A	2.26 (19)	N2B—C9B—C14B—C16B	1.5 (2)
C12A—C13A—C14A—C9A	−0.5 (2)	C12B—C13B—C14B—C9B	−0.9 (2)
C12A—C13A—C14A—C16A	178.65 (13)	C12B—C13B—C14B—C16B	178.56 (14)
C2A—C3A—C17A—C20A	−10.1 (2)	C4B—C3B—C17B—C19B	4.6 (2)
C4A—C3A—C17A—C20A	172.02 (15)	C2B—C3B—C17B—C19B	−174.13 (14)
C2A—C3A—C17A—C18A	109.54 (17)	C4B—C3B—C17B—C18B	123.81 (15)
C4A—C3A—C17A—C18A	−68.33 (18)	C2B—C3B—C17B—C18B	−54.90 (18)
C2A—C3A—C17A—C19A	−130.29 (15)	C4B—C3B—C17B—C20B	−116.55 (15)
C4A—C3A—C17A—C19A	51.83 (19)	C2B—C3B—C17B—C20B	64.74 (17)

### Hydrogen-bond geometry (Å, º)

**Table:**

*D*—H···*A*	*D*—H	H···*A*	*D*···*A*	*D*—H···*A*
N2*A*—H2*NA*···O1*A*	0.84 (2)	2.06 (2)	2.6972 (19)	133.1 (16)
N2*B*—H2*NB*···O1*B*	0.84 (2)	2.08 (2)	2.7183 (19)	132.4 (17)
N2*A*—H2*NA*···S1*B*	0.84 (2)	2.715 (17)	3.2598 (12)	124.3 (16)
N2*B*—H2*NB*···S1*A*^i^	0.84 (2)	2.780 (19)	3.3044 (12)	121.9 (16)

Symmetry code: (i) *x*, −*y*+3/2, *z*+1/2.
